# Air Disinfection—From Medical Areas to Vehicle

**DOI:** 10.3389/fpubh.2022.820816

**Published:** 2022-02-24

**Authors:** Anna Bukłaha, Anna Wieczorek, Ewelina Kruszewska, Piotr Majewski, Dominika Iwaniuk, Paweł Sacha, Elzbieta Tryniszewska, Piotr Wieczorek

**Affiliations:** ^1^Department of Microbiological Diagnostics and Infectious Immunology, Medical University of Białystok, Białystok, Poland; ^2^Department of Infectious Diseases and Neuroinfections, Medical University of Białystok, Białystok, Poland

**Keywords:** car, disinfection, didecyldimethylammonium chloride, peracetic acid (PAA), cinnamaldehyde

## Abstract

Cars with air conditioning systems have become the norm, but these systems can be dangerous for human health as a result of the accumulation of different microorganisms, including pathogenic ones, causing severe allergy or inflammation problems. The novel purpose of this study is 2-fold: on the one hand, to test different disinfection agents on a new area, that is, automobile cabins, and on the other, to compare activity in the gas phase of these agents for disinfection of car air conditioning and cabin surfaces. This study shown that tested disinfectant agents dedicated for decontamination medical areas (agent based on peracetic acid and an agent containing didecyldimethylammonium chloride, 2-phenoxyethanol with cinnamaldehyde) can be successfully used for disinfection car air conditioning and cabin surfaces. Both disinfectants were examined in comparison to a commercial “ready-to-use” spray from a local supermarket dedicated to car air conditioning disinfection. Our research found that very effective agents in this regard were acid stabilized by hydrogen peroxide applied by fumigator, and a combination of didecyldimethylammonium chloride, 2-phenoxyethanol, and cinnamaldehyde applied by atomizer. Tested disinfection procedures of car air conditioning significantly influence the quality of cabin air and surfaces by reducing the amount of microorganisms. The comparison of disinfection properties studied agents in the gas phase reveal statistically significant differences between it effect for disinfection car air conditioning and cabin surfaces. Our research found that very effective agents in this regard were acid stabilized by hydrogen peroxide applied by fumigator, and a combination of didecyldimethylammonium chloride, 2-phenoxyethanol, and cinnamaldehyde applied by atomizer. Tested disinfection procedures of car air conditioning significantly influence the quality of cabin air and surfaces by reducing the amount of microorganisms.

## Introduction

Air conditioners are equipment used in modifying air temperature inside buildings and vehicles. The present work focuses on car air conditioning systems, because air conditioning in cars has become the norm (1).

Many individuals spend significant percentages of their lives traveling inside vehicles. Vehicles are used for commuting from home to work, traveling, and pleasure and business activities. For the average person of driving age in the developed world, the automobile has become indispensable to daily life. Furthermore, professional drivers, including taxi drivers, public transportation drivers, and truckers, spend significantly more time inside motor vehicles compared to other individuals (2). Current health research in the area of in-vehicle air has intensified, but no standards have been drawn for automobile indoor air so far.

A car cabin is a specific environment with different surfaces that accumulate a variety of microorganisms. The high humidity and dust particles in car air conditioning systems create conditions conducive to growth of harmful microorganisms (3–5). Several studies have reported that passengers are exposed to different species of microbes and fungi in these indoor environments (2, 6, 7). However, passengers' immune systems play a crucial role in the exposure risk of passengers to airborne microorganisms within air conditioned vehicles. Depending on the immune system, adverse health effects can range from simple irritations, through allergic reactions, to infectious diseases or toxic response (2, 7, 8).

As early as 1987 scientists observed a relationship between bronchial disease and bacterial or fungal proliferation by the operation of air conditioning systems. Moreover, automobile heaters and air conditioning can induce air turbulence, which would suspend microorganisms from floor mats, seat covers, and occupants' clothes, thereby elevating microbiological pollution levels. In most cases, elevated microbiological concentrations occurred between 5 and 15 min after turning on the automobile air conditioning (9, 10).

Bacteria and fungi inside cars can emit organic compounds and affect vehicle cabin air quality. Many of these compounds are not harmful to human health; however, some of them are toxic. People sometimes can verify air quality on the basis of odor in a car cabin. One of the most sensitive odor detectors is the human nose. Perceivable malodor associated with mold is caused by odorous volatile compounds. The effects of odors usually spread faster than irritation and sensitization consequences (11, 12). The human nose is capable of sensing odorous substances at very low concentration levels. Conversely, odor detection is very subjective; thus, people can perceive differently the same smells (12).

There are many ways that airborne fungi affect human health: they can produce infection in humans, they may cause allergy reactions, fungi can be toxigenic, or they may be a determinant factor in inflammatory reactions (8). However, even non-pathogenic species have the ability to act as allergens and mycotoxin producers. Inhalation exposure to mycotoxins can turn dangerous when it follows inhalation of mycotoxin-containing mold or dust particles, because mycotoxins are relatively stable and do not evaporate from the mold spore. The literature data indicate that it is unlikely that a mycotoxin dose breathed in an indoor home, office, or school environment would produce an acute toxic response, even under the models with extreme conditions (13), although sometimes mycotoxins are involved in pathogenesis (14). Once colonized by fungi, automobiles emit different odors or sensitizing products that affect the passengers of the cars. The major factors of fungal colonization are undoubtedly airborne fungal populations and high air humidity (11).

The second group of microorganisms inside vehicles is bacteriaIt has been proven that frequently touched surfaces and infrequently cleaned sites accumulate a higher concentration of biological contamination (15, 16).

Several research works have shown that bacteria in car air conditioning form biofilms that might release into the cabin air different microorganisms, including environmental strains as well as pathogens such as *Legionella*, but we have not found published studies linking cases of legionellosis with infection through the car air conditioning (17, 18). Results of biofilm formation tests by bacteria isolated from car air conditioning revealed that all bacterial species produced biofilms (19, 20). When these microorganisms enter the vehicle cabin air they can produce adverse health effects for the car users. The consequences of this effect may be nose and eye hypersensibility, asthmatic reactions, and allergic inflammation (5). It is a public health issue that viable and non-viable microorganisms adversely affect humans in the cabin environment of automobiles (21).

Car air conditioning systems involve filter systems protecting the passengers from biological and industrial air pollution. However, filters become a source of danger to the health of vehicle users if they are not regularly exchanged in a correct way (19, 22). Car cabin filters are capable of accumulating contaminants, including pollen, fungi, and microbial fragments, such as proteins (23).

To prevent the negative effects of air conditioning through the emission of microorganisms into the vehicle cabin, disinfection may be performed in different ways. Disinfection is a procedure that relies on killing infectious agents on a surface by direct exposure to chemical or physical agents. For example, fumigation decontamination is advantageous for disinfecting the inside of buildings because fumigation agents are easily dispersed, diffuse into difficult to access areas, decontaminate air and surfaces, and are less labor intensive than spray-based products (24). Fumigant consists of high particle densities of small droplets larger than 1 μm suspended in air (25). Fumigation is so effective that sterilization with fogging application is implemented in the food and pharmaceutical industries, at health care facilities, and other large-area decontamination sites (25, 26).

Fogging technology has the advantage over liquid forms of disinfectants in that the fogging particle size allows the use of lower amounts of disinfectant to be effective (25).

The aim of this study was to estimate the level of microorganism contamination before and after disinfection of car air conditioning systems and cabin surfaces by three different disinfectant agents. The evaluation is based on qualitative and quantitative analysis of microorganisms (bacteria and fungi) isolated from the air and surface samples.

## Experimental Procedures

### Vehicle Characteristics

Thirty four air conditioned private vehicles were recruited and tested in our study. Vehicles were randomly selected and studied during the period of 2018 to 2020 in Bialystok (Poland). Cars were tested in the summer period. Air conditioning systems were turned on during the air measurements. Before cabin air testing samples were taken from such cabin surfaces as the dashboard, driver's seat, driver's door, and flooring behind the driver.

### Disinfection Process Characteristics

Three types of air disinfectants were studied in this research. All disinfectants are registered with the Office for Registration of Medicinal Products, Medical Devices, and Biocidal Products.

1. PAA/HPO–Peracetic acid stabilized by hydrogen peroxide, applied with Aerosept fumigator produced by Laboratoires Anios (France). This method is normally used for the last stage of decontamination in hospitals. The product has a smell similar to vinegar. The disinfection taken place in such a way that a peracetic acid stabilized by hydrogen peroxide in the amount of 350 mL was fumigated by Airborne disinfection device in the closed car cabin.
2. DDAC/PHMB–Commercial “ready-to-use” 200 mL spray from a local supermarket based on alcohol, didecyldimethylammonium chloride, polyheksametylenebiguanide and aromatic composition intended for domestic car air conditioning disinfection. The spray smells very intense and it leaves a fresh scent. In accordance with the directions for use, the container was placed on the floor behind the driver‘s seat, the agent was sprayed for about 3 min. with a closed car cabin.
3. DDAC/CA–Didecyldimethylammonium chloride, 2-phenoxyethanol, and cinnamaldehyde applied with an atomizer. This agent is dedicated for maintenance-free surfaces and medical equipment disinfection. The product has a smell similar to cinnamon. The disinfection process took place as follows: 50 mL pack with disinfectant was applied by a one-time-use disinfection device in the closed car cabin, like as DDAC/PHMB.

The cars remained closed with the switched air conditioning (20 min for PAA/HPO and DDAC/CA and 15 min for DDAC/PHMB) at the medium level of air circulation speed in closed mode (3rd or 4th level of fan speed). After the disinfection process the cabins were opened and aired for 30 min.

### Sampling Procedure

Air and surface sampling were conducted before and after disinfection. Bacteria and fungi were sampled from a 25 cm^2^ surface with Rodac contact plates (Oxoid, UK). A plate was pressed to the different surfaces using the Count-Tact Applicator (bioMérieux, France) for 10 s.

The total numbers colony forming units (CFU) of bacteria and fungi were determined by sampling the air with a MAS at 100 l/min for 10 min directly on plates with specific bacterial/fungal medium. The air conditioning system in each car was running at a temperature of 20°C, at average fan speed, and with cabin air recirculating through the air conditioning system. Air samples were taken in the same way before and after the disinfection process.

### Equipment for Sampling

The CFU of bacteria and fungi on the surfaces were tacked with Count-Tact Applicator (pressure 500 g, 10 s). Rodac contact plates were used for investigation of surface microbial pollution on the surface of 25 cm^2^. The plates contained trypticase soy agar (TSA) for bacteria (Oxoid, UK) and Sabouraud agar (Oxoid, UK) for fungi.

Air samples were taken using Microbial Air Monitoring System MAS-100 (Merck, Germany) based on impaction systems, with sampling times of 10 min equivalent to circulation of 1 m^3^ of air. Standard Petri dishes were used for growing microorganisms: with blood agar (COS-Columbia Agar + 5% sheep blood, bioMérieux, France) for bacteria and Sabouraud agar (Oxoid, UK) for fungi. During the experiment, the MAS-100 was placed in the middle of seats in such a way that air flowing from the air conditioning system was directed at the MAS-100 head and Petri dishes, with all doors and windows closed.

### Sample Analysis

Airborne bacterial CFU/m^3^ were determined with blood agar and fungi CFU/m^3^ with Sabouraud agar; surface bacterial CFU/25 cm^2^ were determined with TSA and Sabouraud agar. After aerobic incubation at 35°C for 24–48 h for bacteria and at 30°C for 48–72 h for fungi, the bacterial and fungi colony units on the agar plates were counted. Bacteria were identified by Gram staining (Aqua-med, Poland; denatured alcohol, Hipernet Sp. z o.o., Poland). The number of colony forming units (cfu) was adjusted using Feller's conversion table, and expressed in CFU/m^3^ for air samples.

### Statistical Analysis

Non-parametric statistics were used for hypothesis testing. Kruskal-Wallis one-way analysis of variance and *post hoc* test were used for comparing the three disinfection techniques.

The Wilcoxon signed-rank test was used for comparison of the microbiological pollution of in-cabin air and surfaces before disinfection and after disinfection. Statistical analyses were performed with the STATISTICA software package, version 13.3 (StatSoftPolska Sp. z o.o., Poland). A *p*-value < 0.05 was considered as the level of significance. Results are expressed as median, first (lower) quartile, and third (upper) quartile.

## Results

Comparison of the Biocidal Effect on Air and Surface Samples in the Car Cabin Before and After Application of Three Agents by Different Techniques.

1. In vehicle air: PAA/HPO showed a statistically significant reduction all examined groups of microorganisms, except for Gram-negative bacteria and Gram-positive Rod. In the case of DDAC/PHMB, we did not observe significant statistical differences in the amounts of microorganisms before and after its application. DDAC/CA statistically significantly reduced the number of microorganisms, except for Gram-negative bacteria and Yeasts.
2. Cabin surfaces: after applying PAA/HPO on all examined surfaces, we observed a statistically significant reduction of microorganisms. After the application of DDAC/PHMB, statistically significant differences were noted only in the case of Fungi on the dashboard and on the door. After applying DDAC/CA on all tested surfaces, except seats, we observed statistically significant differences.

The summary of results obtained during studies on air quality before and after application of agents by different techniques in the cabin of vehicles is presented in Table 1 (for air) and Table 2 (for surfaces).

**Table 1:** Median, quartiles and *p*-value of microorganism numbers in vehicle air before and after application of disinfectants (CFU/m^3^), level of statistical significance *p* < 0.05.

	**PAA/HPO**		**DDAC/PHMB**		**DDAC/CA**	
	**Me (Q1–Q3)**	***p*-value**	**Me (Q1–Q3)**	***p*-value**	**Me (Q1–Q3)**	***p*-value**
Bacteria before	7 (5–19)	0.0033	13 (10–22)	0.0665	21 (15–35)	0.005
Bacteria after	1 (0–2)		11 (8–16)		6 (4–8)	
Gram-positive before	2 (0–7)	0.0179	0 (0–10)	0.4445	8.5 (2–14)	0.0926
Gram-positive after	0 (0–0)		6 (1–12)		5 (0–7)	
Gram-negative before	0 (0–0)	0.2733	0 (0–0)	0.1797	1 (0–23)	0.2367
Gram-negative after	0 (0–0)		0 (0–0)		0.5 (0–2)	
Gram-positive rod-shaped before	5 (1–7)	0.0506	6 (3–14)	0.2621	4 (3–8)	0.0218
Gram-positive rod-shaped after	1 (0–3)		2 (2–8)		1 (1–2)	
Fungi before	12 (6–16)	0.0014	5 (4–8)	0.4412	11 (6–23)	0.0128
Fungi after	0 (0–0)		6 (3–7)		4.5 (3–6)	
Yeasts before	0 (0–0)	–	0 (0–0)	0.4184	0.5 (0–2)	0.2075
Yeasts after	0 (0–0)		0 (0–1)		0 (0–1)	
Molds before	12 (6–16)	0.0014	5 (3–8)	0.7597	10 (6–21)	0.0125
Molds after	0 (0–0)		5 (3–7)		4 (2–6)	

*Me, median; Q1, first quartile; Q3, third quartile; PAA/HPO, peracetic acid stabilized by hydrogen peroxide; DDAC/PHMB, didecyldimethylammonium chloride, polyheksametylene biguanide, and aromatic composition; DDAC/CA, didecyldimethylammonium chloride, 2-phenoxyethanol, and cinnamaldehyde*.

**Table 2:** Median, quartiles and *p*-value of number of microorganisms on vehicle surfaces before and after application of disinfectants (CFU/25 cm^2^), level of statistical significance *p* < 0.05.

	**PAA/HPO**		**DDAC/PHMB**		**DDAC/CA**	
	**Me (Q1–Q3)**	***p*-value**	**Me (Q1–Q3)**	***p*-value**	**Me (Q1–Q3)**	***p*-value**
Dashboard bacteria before	26 (18–44)	0.0029	15 (12–45)	0.5048	36 (25–58)	0.0069
Dashboard bacteria after	1 (0–2)		25 (11–40)		13.5 (10-30)	
Dashboard fungi before	17 (4–52)	0.0022	4 (3–11)	0.0178	33 (14–49)	0.0243
Dashboard fungi after	0 (0–0)		1 (1–3)		15.5 (8–40)	
Seats bacteria before	54 (34–62)	0.0014	22 (13–54)	0.328	30.5 (15–53)	0.0366
Seats bacteria after	5 (2–13)		21 (15–43)		22.5 (11–30)	
Seats fungi before	7 (0–10)	0.0144	2 (2–4)	0.1141	6 (3–10)	0.6464
Seats fungi after	0 (0–1)		1 (1–1)		4 (3–8)	
Doors bacteria before	42 (28–51)	0.0014	33 (18–53)	0.328	65.5 (18–92)	0.0093
Doors bacteria after	2 (0–6)		35 (20–41)		27.5 (20–76)	
Doors fungi before	22 (7–33)	0.0014	4 (1–5)	0.0166	6.5 (4–37)	0.0414
Doors fungi after	0 (0–0)		3 (0–4)		6 (2–12)	
Floors bacteria before	51 (35–87)	0.0014	29 (16–78)	1	84.5 (58–112)	0.005
Floors bacteria after	7 (1–17)		27 (18–78)		48 (40–80)	
Floors fungi before	36 (10–48)	0.0018	8 (3–23)	0.2863	30 (17–48)	0.0125
Floors fungi after	0 (0–1)		6 (4–13)		15.5 (11–29)	

*Me, median; Q1, first quartile; Q3, third quartile; PAA/HPO, peracetic acid stabilized by hydrogen peroxide; DDAC/PHMB, didecyldimethylammonium chloride, polyheksametylene biguanide, and aromatic composition; DDAC/CA, didecyldimethylammonium chloride, 2-phenoxyethanol, and cinnamaldehyde*.

**Figure 1:**
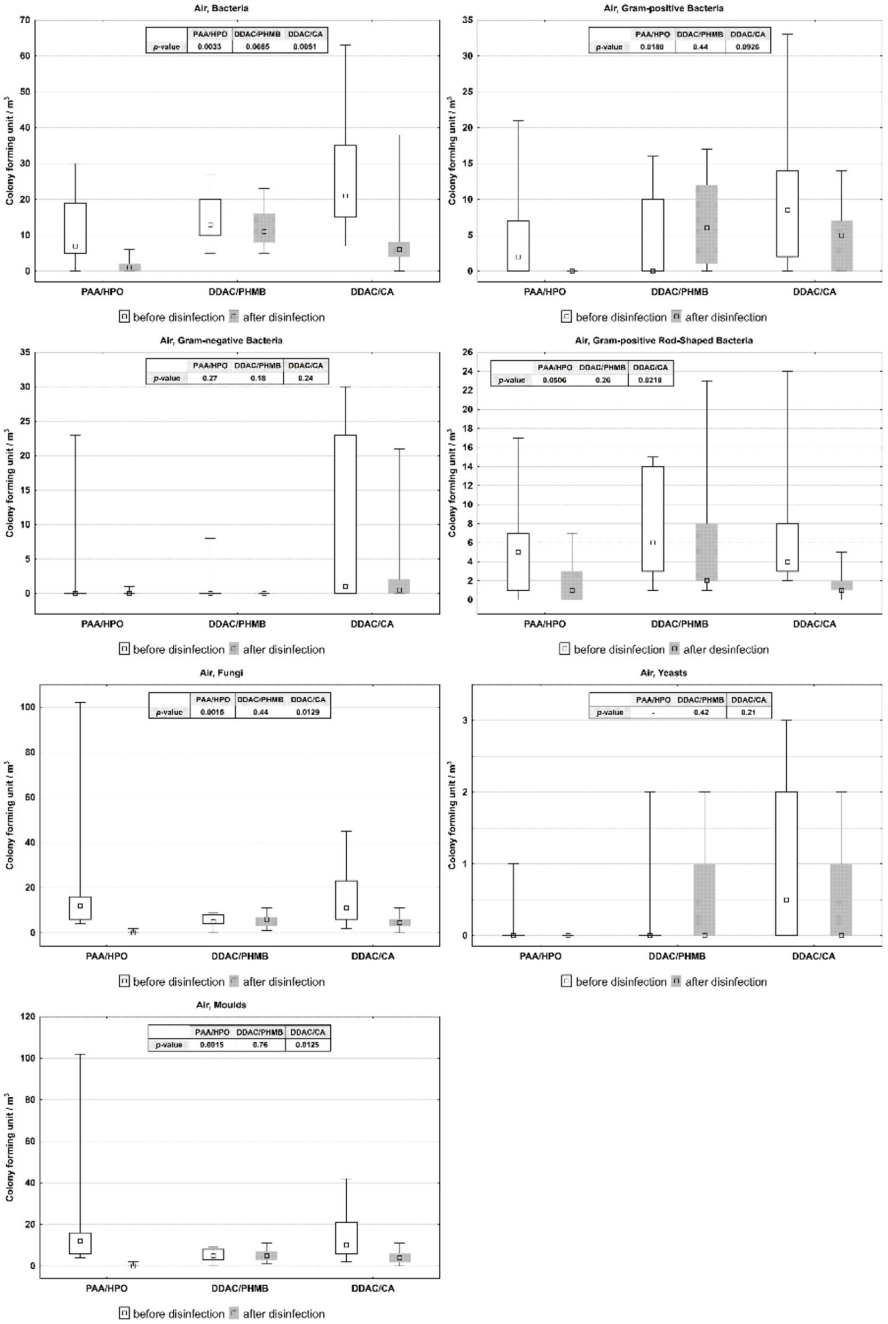
CFU of microorganisms in vehicle air before and after application of disinfectants.

**Figure 2:**
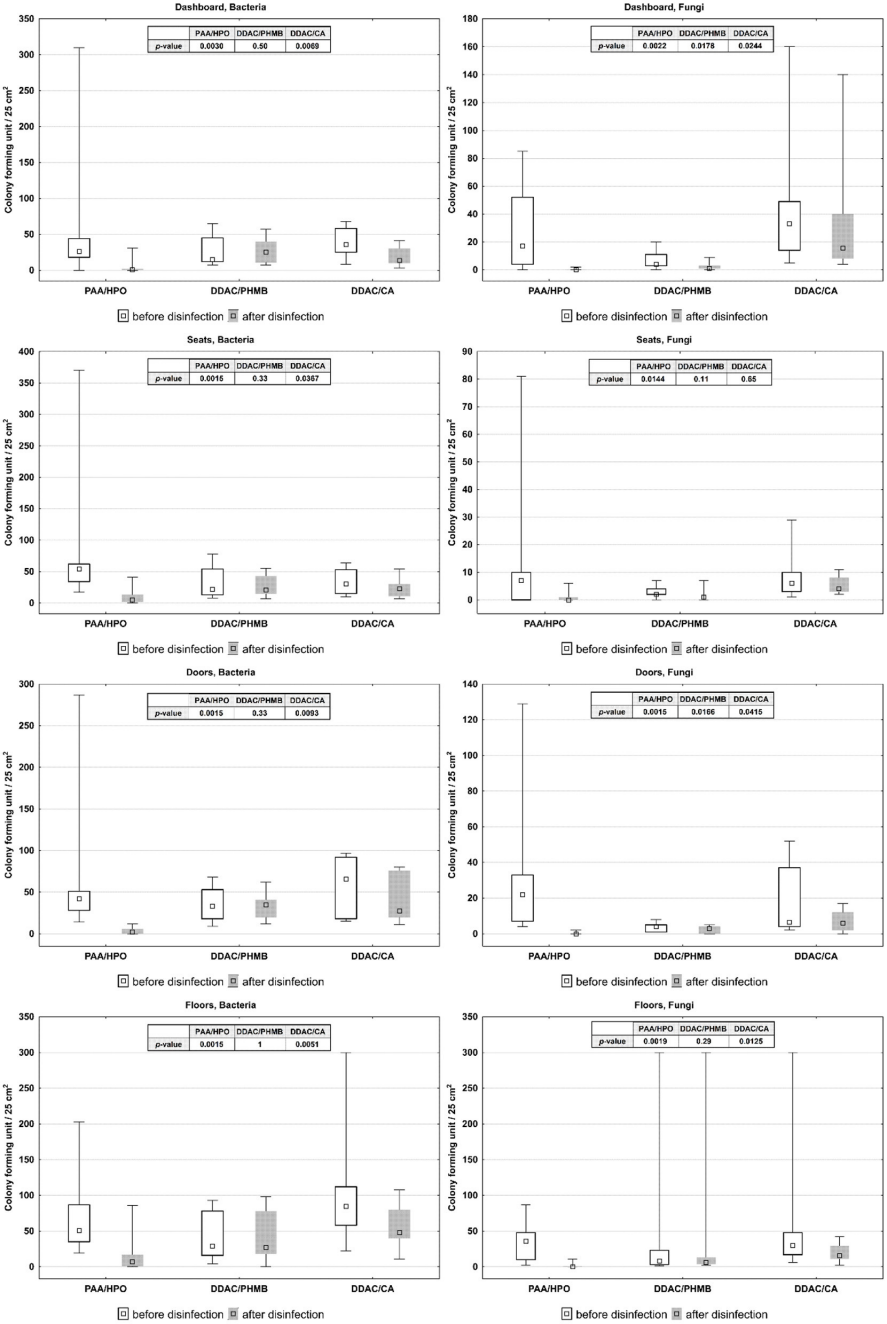
CFU of microorganisms on vehicle surfaces before and after application of disinfectants.

Obtained data were evaluated using graphical method, box-plots showing the results of the Wilcoxon test in Figure 1 (for air) and Figure 2 (for surfaces).

The Comparison of Three Disinfection Technics and Their Effects on Air and Surface Samples in Car Cabin.

1. The comparison of the effectiveness of three disinfection technics on air from car cabins reveal statistically significant differences between the effects of agents DDAC/PHMB - DDAC/CA on bacteria and agents PAA/HPO - DDAC/PHMB and DDAC/PHMB - DDAC/CA on fungi. In other cases the differences in the effects of the three disinfecting agents were statistically insignificant.
2. The comparison of the effectiveness of three disinfection technics on surfaces show statistically significant differences between the effects of agents PAA/HPO - DDAC/PHMB on all microorganisms on surfaces, except Fungi on seats; statistically significant differences between the effects of agents DDAC/PHMB - DDAC/CA on dashboard Bacteria and floors Bacteria and PAA/HPO - DDAC/CA on seats Bacteria. In other cases the differences were statistically insignificant.

Using the Kruskal-Wallis test, the disinfection effects of three agents on the air from air conditioning systems and on in-cabin surfaces were compared. The effects of three disinfectants on vehicle air and surfaces are shown in Table 3 (for air) and Table 4 (for surfaces).

**Table 3:** Median, quartiles and *p*-value of differences among disinfectants affecting the air (CFU/m^3^), level of statistical significance *p* < 0.05.

**Air**						
	**PAA/HPO** **Me (Q1–Q3)**	**DDAC/PHMB** **Me (Q1–Q3)**	**DDAC/CA** **Me (Q1–Q3)**	***post hoc***		
				***p*** **-value**		
				**1-2**	**2-3**	**1-3**
Bacteria	7 (1–16)	2 (0–3)	16.5 (10–23)	0.0799	0.0005	0.2402
Gram-positive	2 (0–7)	−1 (−8 to 2)	4.5 (2-8)	0.2289	0.0976	1
Gram-negative	0 (0–0)	0 (0–0)	0 (−1 to 6)	1	1	1
Gram-positive rod-Shaped	0 (0–6)	2 (−2 to 9)	3 (2–8)	1	0.857	1
Fungi	12 (6–16)	−1 (−3 to 2)	8 (1–14)	0	0.0149	0.6322
Yeast	0 (0–0)	0 (−1 to 0)	0.5 (−1 to 2)	0.8156	0.3991	1
Molds	12 (6–16)	−1 (−3 to 3)	7 (3–11)	0.0001	0.0306	0.5285

*Me, median; Q1, first quartile; Q3, third quartile; PAA/HPO, peracetic acid stabilized by hydrogen peroxide; DDAC/PHMB, didecyldimethylammonium chloride, polyheksametylene biguanide, and aromatic composition; DDAC/CA, didecyldimethylammonium chloride, 2-phenoxyethanol, and cinnamaldehyde*.

**Table 4:** Median, quartiles and *p*-value of differences among disinfectants affecting surfaces (CFU/25 cm^2^), level of statistical significance *p* < 0.05.

	**PAA/HPO** ** Me (Q1–Q3)**	**DDAC/PHMB** ** Me (Q1–Q3)**	**DDAC/CA** ** Me (Q1–Q3)**	***post hoc***		
				***p*** **-value**		
				**1-2**	**2-3**	**1-3**
Dashboard bacteria	22 (16–43)	3 (−3 to 7)	18.5 (9–28)	0.0026	0.0171	1
Dashboard fungi	16 (4–52)	3 (0–4)	7.5 (1–20)	0.031	0.7234	0.6016
Seats bacteria	45 (27–56)	2 (−6 to 11)	8 (3–27)	0.0001	0.8928	0.0118
Seats fungi	7 (0–8)	1 (0–2)	0 (−2 to 5)	0.4849	1	0.314
Doors bacteria	41 (26–47)	6 (−3 to 18)	16,5 (4–31)	0.0005	0.5705	0.0686
Doors fungi	22 (7–33)	1 (1–2)	3 (2–25)	0.0001	0.2623	0.073
Floors bacteria	50 (25–76)	4 (−10 to 9)	29 (18–41)	0.0001	0.0091	1
Floors fungi	36 (9–47)	0 (−2to 6)	6 (2–31)	0.0017	0.2319	0.3843

*Me, median; Q1, first quartile; Q3, third quartile; PAA/HPO, peracetic acid stabilized by hydrogen peroxide; DDAC/PHMB, didecyldimethylammonium chloride, polyheksametylene biguanide, and aromatic composition; DDAC/CA, didecyldimethylammonium chloride, 2-phenoxyethanol, and cinnamaldehyde*.

It was found that the data were non-normally distributed, so the Kruskal-Wallis test was used to determine whether there was a significant difference among the effects of the three agents. Significant differences suggested only that at least one group differed from the other groups. Therefore, *post hoc* tests were performed to determine which groups differed from one another.

**Figure 3:**
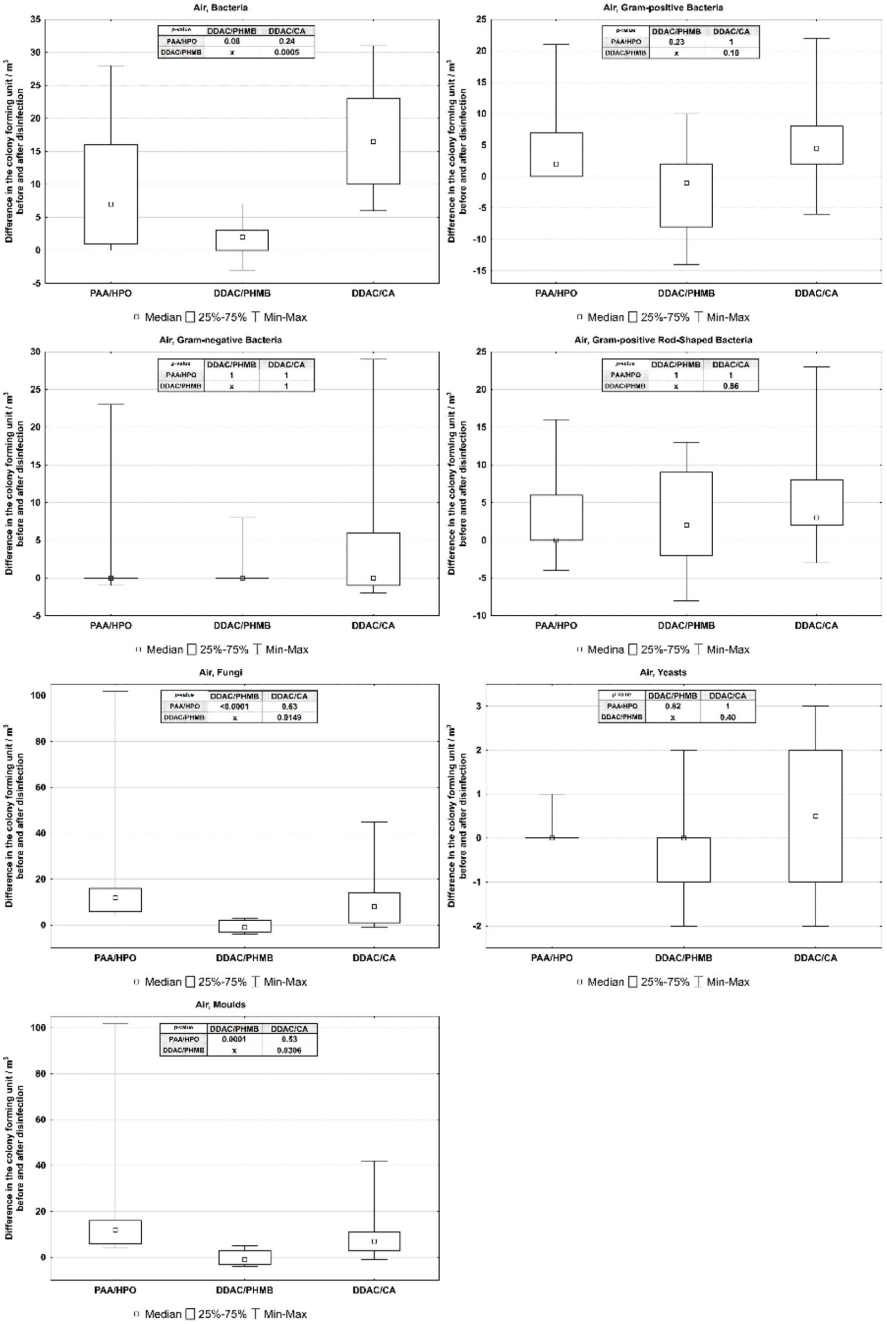
Differences among disinfectants affecting air disinfection.

**Figure 4:**
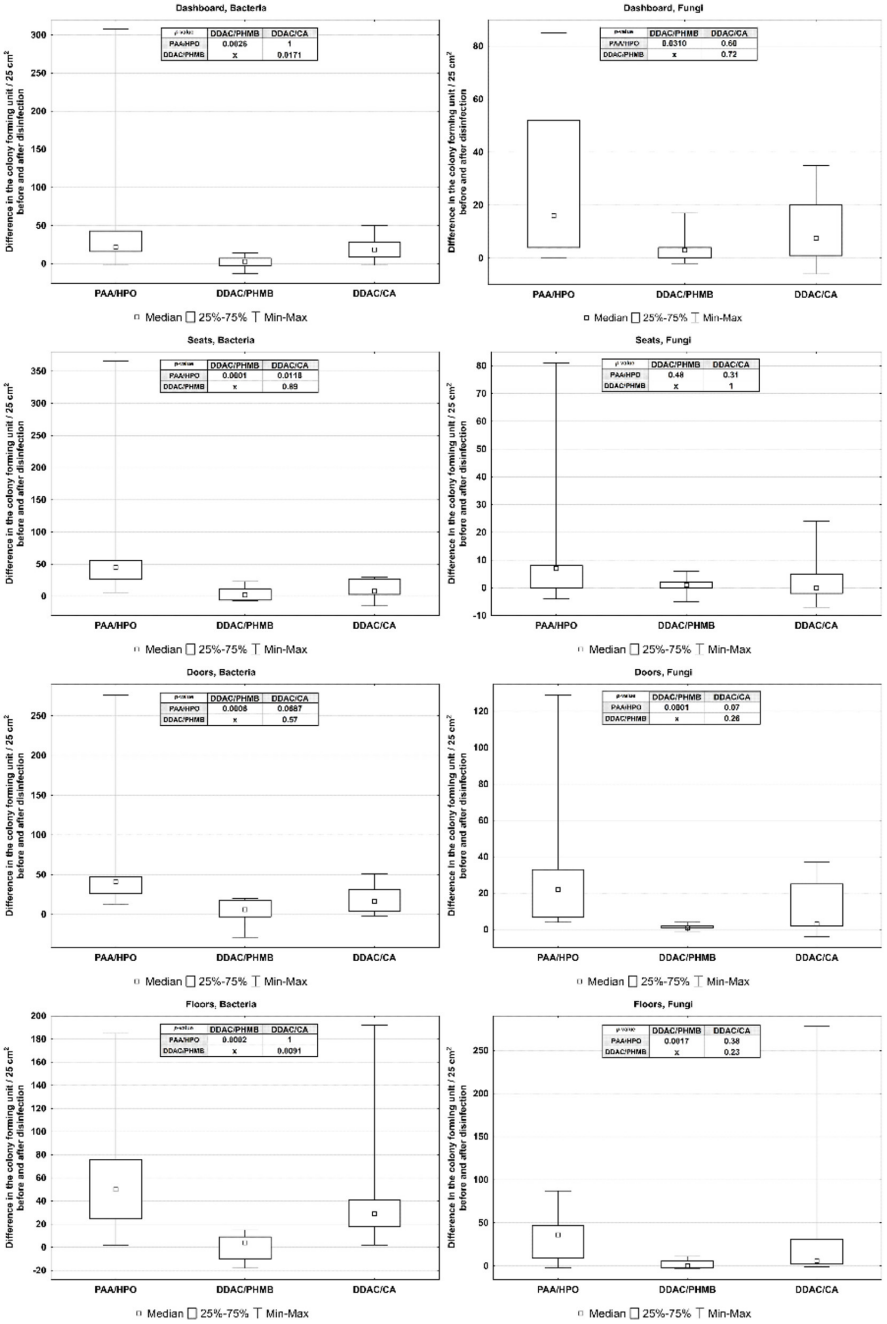
Differences among disinfectants affecting surface disinfection.

Obtained data were evaluated using graphical method, box-plots showing the results of the Kruskal–Wallis test in Figure 3 (for air) and Figure 4 (for surfaces).

## Discussion

The effectiveness of PAA/HPO is demonstrated by the synergistic activity of peracetic acid in combination with hydrogen peroxide. Reports in the literature indicate that the combination of peracetic acid and hydrogen peroxide acts synergistically in comparison to using either agent alone, improving their bactericidal and sporicidal activities (27, 28). The effectiveness of peracetic acid and hydrogen peroxide when used separately is profoundly different, although the substances share mechanisms of action consisting of chemical oxidation of cellular components (29, 30). The advantage of PAA/HPO is its high effectiveness and eco-friendly properties, because its decomposition products are water, oxygen, and carbon dioxide. However, it can be aggressive for metal equipment parts, causing corrosion and leaving an intense vinegar smell after disinfection.

DDAC/PHMB produces a didecyldimethylammonium chloride effect, because this compound is a membrane-active agent resulting in disinfectant properties (31). Literature data have shown that didecyldimethylammonium chloride–based disinfectants produced significant activity against Gram-positive bacteria and were ineffective against Gram-negative strains. Due to its antimicrobial effect and its low irritation, corrosiveness, and toxicity, didecyldimethylammonium chloride is used in healthcare, veterinary, and food facilities (32). However, our research showed no statistical significance differences between the number of bacteria and fungi before and after air and surface disinfection. Our results suggested an ineffectiveness by DDAC/PHMB application on the studied microorganisms in-vehicle.

The disinfection properties of DDAC/CA are the result of the combination of didecyldimethylammonium chloride and cinnamaldehyde. The cinnamaldehyde component is characterized by anti-inflammatory, antioxidant, and antimicrobial properties (33). Cinnamaldehyde can be used in air care products, perfumes, waxes, washing and cleaning products, cosmetics, personal care and household products, pharmaceuticals, and biocides. A higher reduction of microorganisms was observed when biocide containing a combination of didecyldimethylammonium chloride with cinnamaldehyde in comparison with didecyldimethylammonium chloride alone was used. This synergistic effect between a biocidal product and cinnamaldehyde was previously known to scientists (34).

The low level of disinfection observed on seats could be caused by the soft surface of the seats, which reduces the disinfecting effect, because many small holes and gaps in the material allow pathogens to hide.

The use of a combination of disinfectant and cinnamaldehyde with didecyldimethylammonium chloride to improve efficiency in air and surface disinfection in-vehicle is also approved for food use, as it is harmless for humans in addition to leaving a pleasant cinnamon flavour. It was noted for the vehicles that received fragrance in our study that disinfectant with cinnamaldehyde reduced bad odours. An additional advantage of using a biocide in combination with phytochemical is reducing the concentration of synthetic disinfectants and its negative environmental and public health impacts (34).

## Conclusions

Our study presented evidence that disinfection of car environments is very important for users to prevent the health risks associated with using air conditioning systems. Neglect or over-used car air conditioning can result in dust and microorganism accumulations. For this reason, vehicle users are exposed to different harmful species. Disinfection procedures of car air conditioning significantly influence the quality of cabin air and surfaces by reducing the amount of microorganisms. Our research found that very effective agents in this regard were acid stabilized by hydrogen peroxide applied by fumigator, and a combination of didecyldimethylammonium chloride, 2-phenoxyethanol, and cinnamaldehyde applied by atomizer. PAA/HPO it is an effective disinfectant but requires the use of a specialized device, peracetic acid is aggressive toward metal surfaces and leaves intense vinegar smell. On the other hand, the DDAC/CA is also effective, does not require any additional device and leaves a pleasant cinnamon scent.

With frequent use of car air conditioning, it is important to practice regular maintenance; otherwise, car drivers and passengers may be exposed to the unhealthy influence of microorganisms, which is especially dangerous in the context of COVID-19 pandemic.

## Data Availability Statement

The original contributions presented in the study are included in the article/supplementary material, further inquiries can be directed to the corresponding author/s.

## Author Contributions

AB: research concept and design, collection of data, data analysis and interpretation, and writing the article. AW and EK: collection of data and data analysis. PM: data analysis and interpretation. DI: collection of data. PS and ET: critical revision of the article. PW: research concept and design and final approval of article. All authors contributed to the article and approved the submitted version.

## Funding

This work was supported by the Project entitled National intersectoral doctoral studies at the Medical University of Bialystok (POWR.03.02.00-00-I050/16) co-funded from European Union funds within the framework of European Social Fund as part of Knowledge Education Development 2014-2020 Operational Programme, through the grant no 01/MSD/2019.

## Conflict of Interest

The authors declare that the research was conducted in the absence of any commercial or financial relationships that could be construed as a potential conflict of interest.

## Publisher's Note

All claims expressed in this article are solely those of the authors and do not necessarily represent those of their affiliated organizations, or those of the publisher, the editors and the reviewers. Any product that may be evaluated in this article, or claim that may be made by its manufacturer, is not guaranteed or endorsed by the publisher.

## References

[1] Barnes NM Ng TW Ma KK Lai KM. In-cabin air quality during driving and engine idling in air-conditioned private vehicles in Hong Kong. Int J Environ Res and Public Health. (2018) 15:611. 10.3390/ijerph15040611 29584686 PMC5923653

[2] Gołofit-Szymczak M Stobnicka-Kupiec A Górny RL. Impact of air-conditioning system disinfection on microbial contamination of passanger cars. Air Qual Atmos Health. (2019) 12:1127–35. 10.1007/s11869-019-00731-7

[3] Grzybowski P. Ocena I kontrola czystości mikrobiologicznej powietrza w samochodowych układach klimatyzacyjnych. Inz Ap Chem. (2011)50:34–5.

[4] Anas G Aligbe DS Suleiman G Warodi FA. Studies on microorganisms associated with air-conditioned environments. J Env Sci. (2016) 10:16–8. 10.9790/2402-1007011618

[5] Gołofit-Szymczak M Górny RL Ławniczek-Wałczyk A Stobnicka A Cyprowski M Bakal A. Automobile air-conditioning systems as a source of microbial contaminants. Ecol Safety. (2017) 11:165–72.

[6] Lee J-H Jo W-K. Exposure to airborne fungi and bacteria while commuting in passenger cars and public buses. Atmosph Environ. (2005) 39:7342–50. 10.1016/j.atmosenv.2005.09.013

[7] Gołofit-Szymczak M Stobnicka-Kupiec A Górny RL Cyprowski M Ławniczek-Wałczyk A. Microbial air quality in municipal buses before and after disinfection of their air-conditioning systems. J Ecol Eng. (2019) 20:189–94. 10.12911/22998993/113408

[8] Udaya Prakash NK Bhuvaneswari S Ranjith Kumar M Lankesh S Rupesh K. A study on the prevalence of indoor mycoflora in air conditioned buses. Br Microb Res J. (2013) 4:282–92. 10.9734/BMRJ/2014/5380

[9] Jo W-K Lee J-H. Airborne fungal and bacterial levels associated with the use of automobile air conditioners or heaters, room air conditioners, and humidifiers. Arch Environ Occup Health. (2008) 63:101–7. 10.3200/AEOH.63.3.101-107 18980872

[10] Li J Li M Shen F Zou Z Yao M Wu C. Characterization of biological aerosol exposure risks from automobile air conditioning system. Environ Sci Technol. (2013) 47:10660–6. 10.1021/es402848d 23952908

[11] Simmons RB Noble JA Rose L Price DL Crow SA Aheam DG. Fungal colonization of automobile air conditioning systems. J Industr Microbiol Biotechnol. (1997) 19:150–3. 10.1038/sj.jim.2900451

[12] Faber J Brodzik K. Air quality inside passenger cars. AIMS Environ Sci. (2017) 4:112–33. 10.3934/environsci.2017.1.112

[13] Kelman BJ Robbins CA Swenson LJ Hardin BD. Risk from inhaled mycotoxins in indoor office and residential environments. Int J Toxicol. (2003) 23:3–10. 10.1080/10915810490265423 15162841

[14] Fischer D Dott W. Relevance of airborne fungi and their secondary metabolites for environmental, occupational and indoor hygiene. Arch Microbiol. (2003) 179:75–82. 10.1007/s00203-002-0495-2 12560984

[15] Otter JA French GL. Bacterial contamination on touch surfaces in the public transport system and in public areas of a hospital in London. Lett Appl Microbiol. (2009) 49:803–5. 10.1111/j.1472-765X.2009.02728.x 19818007

[16] Stephenson RE Gutierrez D Peters C Nichols M Boles BR. Elucidation of bacteria found in car interiors and strategies to reduce the presence of potential pathogens. Biofouling. (2014) 30:337–46. 10.1080/08927014.2013.873418 24564823 PMC3962071

[17] Gołofit-Szymczak M Stobnicka-Kupiec A. Mikrobiologiczna jakość powietrza w klimatyzowanych samochodach osobowych. Ann Set Environ Protect. (2018) 20:1564–82.

[18] Sattar SA Wright KE Zargar B Rubino JR Khalid Ijaz M. Airborne infectious agents and other pollutants in automobiles for domestic use: potential health impacts and approaches to risk mitigation. J Environ Public Health. (2016) 2016:2–12. 10.1155/2016/1548326 28042302 PMC5155087

[19] Vonberg R-P Gastmeier P Kenneweg B Holdack-Janssen H Sohr D Chaberny IF. The microbiological quality of air improves when using air conditioning systems in cars. BMC Infect Dis. (2010) 10:146. 10.1186/1471-2334-10-146 20515449 PMC2890006

[20] Rahman S. Detection of bacterial population in air conditioner and determine the ability to produce biofilm. Iraqi J Sci. (2019) 60:432–7. 10.24996/ijs.2019.60.3

[21] Sowiak M Kozajda A Jezak K Szadkowska-Stanczyk I. Does the air conditio system in busses spread allergic fungi into driver space? Environ Sci Poll Res. (2017) 25:5013–23. 10.1007/s11356-017-0830-4 29209965 PMC5846988

[22] Fiegel J Clarke R Edwards D. Airborne infectious disease and the suppression of pulmonary bioaerosols. Drug Discov Today. (2016) 11:51–7. 10.1016/S1359-6446(05)03687-1 16478691 PMC7108402

[23] Hurley KV Wharton L Wheeler MJ Skjoth CA Niles C Hanson MC. Car cabin filters as sampling devices to study bioaerosols using eDNA and microbiological methods. Aerobiologia. (2019) 35:215–25. 10.1007/s10453-018-09554-y

[24] Meyer KM Calfee MW Wood JP Mickelsen L Attwood B Clayton M. Fumigation of a laboratory-scale HVAC system with hydrogen peroxide for decontamination following a biological contamination incident. J Appl Microbiol. (2013) 116:533–41. 10.1111/jam.12404 24279292

[25] Hayrapetyan H Nederhoff L Vollebregt M Mastwijk H Groot MN. Inactivation kinetics of *Geobacillus stearothermophilus* spores by a peracetic acid or hydrogen peroxide fog in comparison to the liquid form. Int J Food Microbiol. (2020) 316:1–9. 10.1016/j.ijfoodmicro.2019.108418 31877424

[26] Richter WR Wood JP Wendling MQS Rogers JV. Inactivation of Bacillus anthracis spores to decontaminate subway railcar and related materials via the fogging of peracetic acid and hydrogen peroxide sporicidal liquids. J Environ Manage. (2018) 206:800–6. 10.1016/j.jenvman.2017.11.027 29174643 PMC5738270

[27] Boyce JM. Modern technologies for improving cleaning and disinfection of environmental surfaces in hospitals. Antimicrob Resist Infect Control. (2016) 5:1–10. 10.1186/s13756-016-0111-x 27069623 PMC4827199

[28] Leggett MJ Schwarz S Burke PA McDonnell G Denyer SP Maillard J-Y. Mechanism of sporicidal activity for the synergistic combination of peracetic acid and hydrogen peroxide. Appl Environ Microbiol. (2016) 82:1035–9. 10.1128/AEM.03010-15 26637595 PMC4751845

[29] Finnegan M Linley E Denyer SP McDonnell G Simons C Maillard J-Y. Mode of action ofhydrogen peroxide and other oxidizing agents: differences between liquid and gas forms. J Antimicrob Chemother. (2010) 65:2108–15. 10.1093/jac/dkq308 20713407

[30] Korany AM Hua Z Green T Hanrahan I El-Shinawy SH El-kholy A. Efficacy of ozonated water, chlorine, chlorine dioxide, quaternary ammonium compounds and peroxyacetic acid against *Listeria monocytogenes* biofilm on polystyrene surfaces. Front Microbiol. (2018) 9:2296. 10.3389/fmicb.2018.02296 30369909 PMC6194171

[31] Ioannou CJ Hanlon GW Danyer SP. Action of disinfectant quaternary ammonium compounds against *Staphylococcus aureus*. Antimicrob Agents Chemother. (2007) 51:296–306. 10.1128/AAC.00375-06 17060529 PMC1797692

[32] Montagna MT Triggiano F Barbuti G Bartolomeo N Giglio O Diella G. Study on the in vitro activity of five disinfectants againstnosocomial bacteria. Int J Environ Res Public Health. (2019) 16:1–9. 10.3390/ijerph16111895 31146343 PMC6603693

[33] Fabra MJ Castro-Mayorga JL Randazzo W Lagarón JM López-Rubio A Aznar R. Efficacy of cinnamaldehyde against enteric viruses and activity after incorporation into biodegradable multilayer systems of interest in food packing. Food Environ Virol. (2016) 8:125–32. 10.1007/s12560-016-9235-7 27008344

[34] Malheiro JF Oliveira K Cagide F Borges F Simoes M Maillard J-Y. Surface wiping test to study biocide-cinnamaldehyde combination to improve efficiency in surface disinfection. Int J Mol Sci. (2020) 21:1–14. 10.3390/ijms21217852 33113903 PMC7660177

